# Weaponizing the digital divide: a Weberian analysis of elite domination in Indonesia political contestation

**DOI:** 10.3389/fsoc.2026.1866231

**Published:** 2026-07-30

**Authors:** Reza Amarta Prayoga, Erlita Tantri, Arditya Wicaksono, Eko Wahyono, Djoko Puguh Wibowo, Cahyo Pamungkas, Rita Pawestri Setyaningsih, Devi Tri Indriasari

**Affiliations:** 1 The National Research and Innovation Agency, Central Jakarta, Indonesia; 2 Department of Sociology, Faculty of Social and Political Sciences, University of Indonesia, Kota Depok, Indonesia

**Keywords:** algorithmic charisma, digital divide, new digital media, political contestation, post-truth, Weberian

## Abstract

**Introduction:**

Although Max Weber did not explicitly address digital technology, his theoretical framework remains crucial for understanding contemporary media power. In transitional democracies like Indonesia, widespread internet connectivity contrasts sharply with low national digital literacy, shifting the “digital divide” from a primary crisis of infrastructural access to a secondary crisis of cognitive competence. This study examines how political elites weaponize this secondary divide within the post-truth landscape to dominate the public attention space and consolidate power.

**Methods:**

Grounded in Weberian digital sociology, this qualitative study utilized a triangulated research design combining a systematic literature review (*n* = 42), a documentary study of legislative and party archives (*n* = 25), and a six-month digital observation (October 2023 – March 2024) across social media platforms (X and Instagram) during the 2024 Indonesian Presidential Election. Data were analyzed using deductive thematic coding to trace algorithmic rationalization and engagement mechanics.

**Results:**

The findings reveal that low digital literacy creates a profound cognitive vacuum that elites exploit through automated digital bureaucracies. By deploying AI-generated campaign strategies—most notably the synthetic “Gemoy” avatar during the 2024 election—political elites bypassed traditional institutional gatekeepers. This dynamic manifested a modernized form of plebiscitary charismatic domination driven by three empirical mechanisms: *algorithmic satiation*, *cognitive desensitization*, and *procedural validation*.

**Discussion:**

This study advances Weberian scholarship by introducing the concepts of “Algorithmic Charisma”—where emotional authority is synthetically manufactured via machine learning—and the “Participatory Iron Cage,” wherein hyper-connected users voluntarily reproduce elite-driven platform metrics. Ultimately, the digital divide in the Global South functions not merely as a metric of socio-economic inequality, but as a rationalized structural weapon of elite political hegemony.

## Introduction

The evolution of contemporary digital media has fundamemtally restructured teh asymmetric power dynamic between political elites and society. In his seminal comparative historical analysis of information and communication technology (ICT) system across the United States, Sweden, China, and India, Schroeder (2015) underscores how variations in media infrastructure are deeply intertwined with distinct cultural pluralities and political regime, ranging froma autonomous market-driven structures to strict state-controlled censorship. Although Max Weber never addressed news media or digital technology during his lifetimes (Darmon and Frade, 2013; Schroeder, 2015), his theoretical framework remains recognized as a foundational pillar of contemporary social science (Weber, 1988). Schroeder (2015) application of Weberian thought effectively connect structural technological shifts with macro-sociological issues, particularly by emphasizing how the relationship between elites and society is actively mediated by the media. This analytical lens highlight how two crutial dynamics, (1) the political and cultural utilization of media systems to sustain elite-society relations; and (2) the structural role of elites in driving consumer culture (Schroeder, 2007, 2015). Within this framework, political elites exert a highly sophisticated control over political content, which is continuously reproduced and reinforced by digital media through regulated yet fundamentally unbalanced asymmetries (Schroeder, 2015).

Weber’s sociology posits that social structures are inherently shaped by dominant belief systems, which serve as the primary arena where elites construct, negotiate, and legitimize their status. In contemporary society, these dominant belief are increasingly mediated by advanced information technologies, transforming popular culture into what Kurylo (2020) conceptualizes as a technologized consumer culture, where both popular culture and elite engage in rigorous status differentiation (Schroeder, 2007, 2015). Reed (2018), Ahen (2019), and Ben Lagha et al. (2026) identifies this profound interaction between digital platforms and emerging device as a defining characteristic of the contemporary ‘post-truth’ era, wherein technological sophistication systematically blurs the boundaries between empirical reality and public perception. This digital mediation induces a severe crisis in dominat belief systems, as the foundational parameters of truth and validity are distorted by global digital architectures often manipulated by a select elite (Reed, 2018). Consequently, within the contemporary political order, “what is right can appear wrong, and what is wrong can appear right” on social media platforms, fundamentally disrupting the traditional political sphere where established professional media elites historically preserved narratives of institutional trust and systemic belief in alignment with state authorities.

However, a profound paradox arises in transitional democracies like Indonesia. While the country has experienced a massive expansion in internet connectivity and boasts one of the world’s largest cohorts of active social media users, this rapid technological penetration has not been accompanied by a corresponding growth in digital competence. This discrepancy exposes a critical shift in the nature of the digital divide: it has evolved from a primary crisis of infrastructural access into a secondary crisis of digital literacy, ethics, and critical information processing. In this systemic landscape, low digital literacy renders vast segment of the population highly vulnerable to algorithmic manipulation, misinformation, and targeted hoaxes.

While conventional media studies in Indonesia frequently rely on Neo-Marxist political economy frameworks to critique media conglomeration and capital ownership, they often overlook the micro-sociological mechanisms of algorithmic rationalization and platformization. There is a noticeable academic gap in explaining how structural deficits in digital competence are actively converted into political capital. Current literature has yet to adequately theorize how political elites strategically adapt to decentralized platforms to bypass traditional institutional gatekeepers and systematically dominate the public “attention space”.

To address this empirical and theoretical gap, this paper examines how the digital divide operates within digital media and explores the social consequences of digital media elites’ power in shaping political contestation in Indonesia. By synthesizing a Weberian perspective of rationalized bureaucracy with the dynamics of contemporary platformization, this study offers a twofold academic contribution. First, it redefines the digital divide not merely as a technical problem of socio-economic access, but as a structural weapon of elite political domination in the post-truth era. Second, it provides empirical grounding to this theoretical framework by dissecting the algorithmic packaging of political campaigns in the 2024 Indonesian Presidential Election, demonstrating how elite control over digitized content systematically shapes rational public opinion and manufactures collective decision-making.

## Method

This study adopts a qualitative research design grounded in a Weberian sociological framework to investigate how the digital divide in Indonesia has been structurally weaponized as an instrument of elite political domination. To mitigate the limitations of single-method biases and rigorously enhance the structural validity and replicability of the findings, a triangulated analytical approach was established. This design systematically integrates a systematic literature review (SLR) to map macro-structural boundaries, a documentary study of institutional archives to trace formal political narratives, and direct digital observation of social media spaces to capture real-time power dynamics during high-stakes political contestation.

The first component, the systematic literature review, was executed to trace the conceptual evolution of the digital divide from a primary issue of infrastructural access to a secondary crisis of cognitive, ethical, and cultural competence. The selection protocol was bound by a strict timeframe targeting academic journals, peer-reviewed articles, and institutional reports published between 2020 and 2025. Database searches were comprehensively carried out across prominent global and national indices, including Scopus, Web of Science, Google Scholar, and the Science and Technology Index (Sinta) to capture localized empirical realities. Utilizing precise Boolean search strings such as “digital divide Indonesia,” “Weberian digital media,” “elite political domination,” and “algorithmic power,” the initial query yielded a preliminary pool of 85 sources. Through rigorous abstract screening and relevance filtering based on their explicit theoretical alignment with Weberian sociology and empirical focus on the Indonesian digital landscape, a finalized sample of 42 high-impact academic works was retained for core analytical synthesis.

Complementing the literature review, the documentary study served as the primary mechanism for structural verification, treating formal political and legislative texts as “texts of power” embedded with indicators of rational-legal authority and bureaucratization. The corpus of analyzed documents comprised 15 official press releases from prominent Indonesian political parties, 10 legislative digital archives regarding national information technology, digital literacy initiatives, and cybersecurity regulations, and the verified social media communications issued by the primary presidential candidates of the 2024 election cycle. These documents were subjected to systematic thematic coding to uncover the underlying operational mechanisms through which elite interests are institutionalized, rationalized, and subsequently legitimized within the evolving public sphere.

To capture the behavioral manifestations of elite domination and public engagement, digital observation was conducted over a continuous six-month period stretching from October 2023 to March 2024, encapsulating the absolute peak of the 2024 Presidential Election campaign and its immediate post-election atmosphere. The observation was structurally localized on X (formerly Twitter) and Instagram, selected due to their high penetration rates and role as primary arenas of political discourse among active millennial and Gen Z cohorts in Indonesia. The operational focus of this observation rested on tracking the strategic dissemination of AI-generated political content—most notably exemplified by the algorithmic packaging of the “Gemoy” campaign—as well as monitoring specific viral political hashtags, auditing user interaction patterns, and analyzing the micro-politics of public engagement within the comment sections of elite accounts.

Data collected from these three convergent streams were synthesized using a deductive thematic analysis anchored in the Weberian theoretical tradition. The analytical procedure strictly followed three sequential phases: data reduction, data display, and conclusion drawing or verification. During data reduction, raw texts, archive transcriptions, and digital observational logs were systematically filtered to isolate phenomena directly pertaining to digital literacy deficits, personal data breaches, and elite structural campaigning strategies. To ensure high methodogical transparency, our coding procedure was operationalized thourgh a two-tiered qualitative matrix. First, open coding was execute manually to identify recurring linguistic pattern, paltform interactions, and aesthetic tropes across the 15 party press release and social media comment sections. Second, axial coding was deployed to cluster theses raw nodes into our predefined analytical indicators: digital bureaucracy (procedural metric, Ai gatekeeping), rationalized domination (data exploitation, secondary digital divide), and attention space contestation (algorithmic charisma, cognitive desensitization). To guarantee internal validation and eliminate subjective researcher bias during the analysis of a highly polarized election, a peer-debriefing and cross-coding consensus protocol was maintained among all co-authors. Any ambiguities regarding thematic classifications were collectively cross-referenced with Schroeder (2015) comparative bencmarks and empirical data form We Are Social (2023/2024) reports, establishing a multi-layered triangulation that solidifies the structural validity and reproducibility of the study’s conclusions.

## Result

### Anatomy of theoretical aspects: translating Weberian rationalization into digital infrastructure

**Figure 1:**
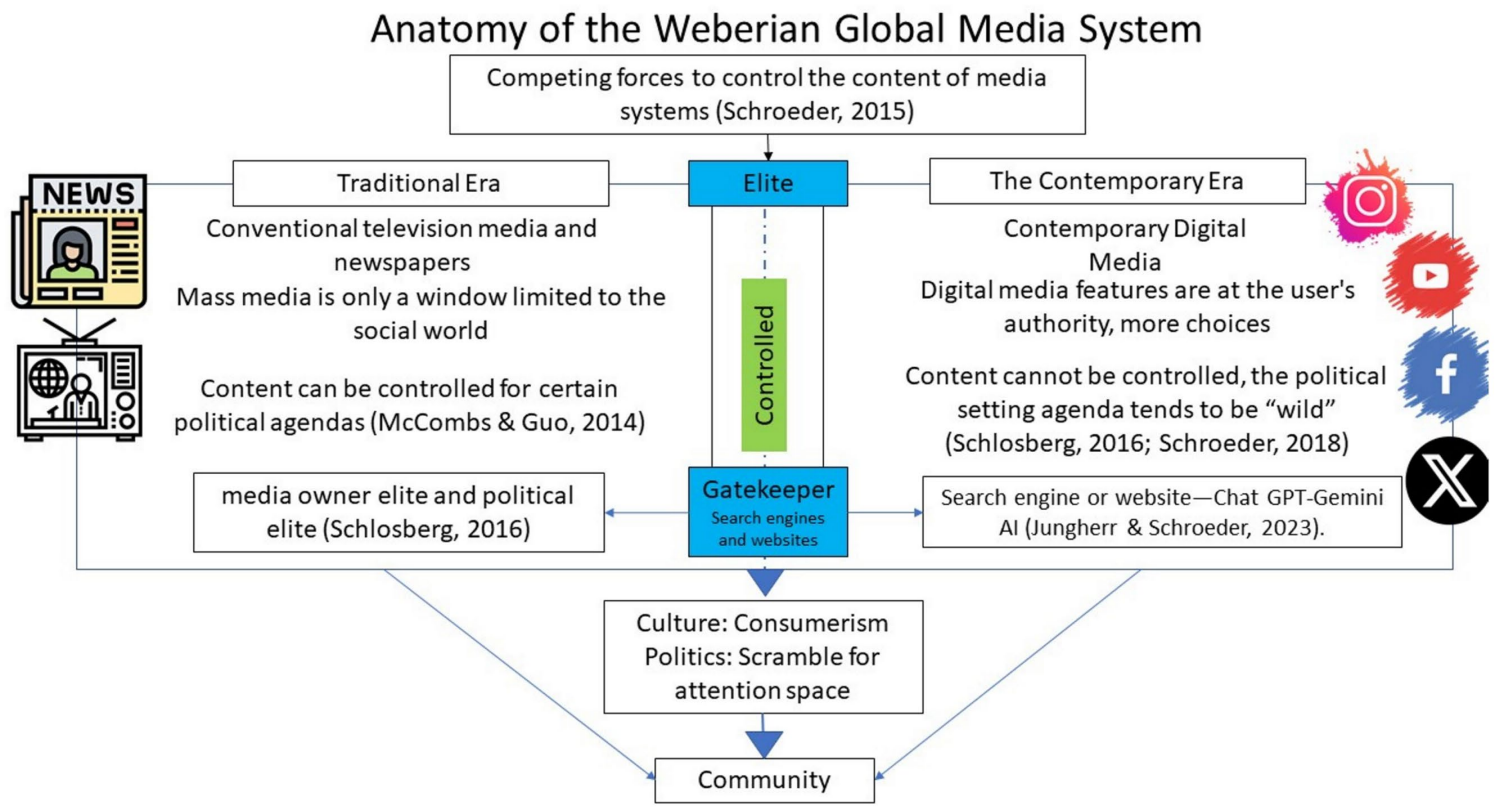
Anatomy of the Weberian global media system. Source: (processed by reviewers from Schroeder’s findings, 2015).

The structural transition from traditional mass media to contemporary social platforms does not signify a democratization of the public sphere; rather, it represents a shift in the mechanisms of bureaucratic control. As illustrated in Figure 1, the conventional era was characterized by centralized, physical gatekeepers—primarily media owners and political elites—who structurally constrained the volume of information to align with specific institutional agendas (McCombs and Guo, 2014; Schlosberg, 2016). Within a Weberian framework, this traditional apparatus operated akin to a classic legal-rational bureaucracy, where information flow was heavily structured through formalized editorial hierarchies. Conversely, the contemporary digital ecosystem shifts authority directly to the user, granting an illusion of autonomy in content selection (Luhmann, 2000). However, our empirical synthesis of current platform architectures demonstrates that control has merely been displaced from human editors to impersonal, algorithmic gatekeepers—such as search engines, algorithmic feeds, and generative artificial intelligence frameworks like ChatGPT and Gemini AI (Jungherr and Schroeder, 2023).

This structural shift manifests what Weber famously critiqued as the “iron cage” (*Stahlhartes Gehäuse*) of rationalization. Contemporary digitalization functions as a highly automated digital bureaucracy where individual user actions, prompts, and behavioral inputs are processed through templated, standardized platforms designed to maximize engagement and optimize information flows (Li et al., 2024). Schroeder (2015) notes that while political elites exert control over content, this dominance operates through asymmetric and unbalanced digital relationships. When juxtaposed with Neo-Marxist models (e.g., Castells) that view media control purely through economic capital and corporate conglomeration, or Durkheimian views on media rituals, the Weberian lens uniquely exposes how platformization subtly reorganizes daily life by substituting substantive rationality (critical, value-driven evaluation of truth) with formal rationality (procedural efficiency, metric maximization, and algorithmic visibility). Elite dominance is thus sustained because the digital medium itself functions as an automated machine, treating users as predictable cogs within an engineered “attention space” (Schroeder, 2015, 2018).

To operationalize this Weberian digital framework and establish an empirically verifiable link between structual inequality and political power, Table 1 outline the precise conceptual definitions and analytical indicators utilized throughout thi study. By translating macro-sociological abstractions into discrete platform mechanisms, this matrix bridge the gap between classic authority and contemporary algorithmica dynamic.

**Table 1:** Conceptual framework and analytical indicators of digital domination.

Core concept	Conceptual definition (Weberian context)	Empirical/analytical indicators in digital space
Elite domination	The capacity of political and digital media elites to pbypas traditional institutional gatekeepers and manufacture public legitimacy through automated, legal-rational procedures.	Coordinated algorithmic amplication of elite agendas. Production of synthetic/AI generated political persona (e.g., automated avatars). Disintermediation of traditional journalistic scrutiny. Structural deployment of state-aligned cyber-troops and informational echo chambers.
Attention space	A compressed visually-driven digital arena where user engagement is systematically engineered to optimize behavioral retention shile minimizing substantive value-rational reflection.	Metric maximation (quantifiable scaling of likes, views, share, and algorithmic trends). Platformization and feeds saturation (e.g., TikTok FYP placement). Structural displacement of complex policy/ideological discourse by aesthetic consumerist gimmicks.
Weaponization of the digital divide	The strategic exploitation of stratified cognitive competence (the secondary digital divide) to convert structural knowledge deficits into uncritical political capital and electoral compliance.	Polarization of platform usage based on users educational backgrounds. Low cognitive indeks in evaluating digital truth (high susceptibility to hoaxes/misinformation). Absence of independent value-rational validation mechanisms among hyper-connected mass audiences. Exploitation of compromised personal data for algorithmic behavioral micro-targeting.

Sources: the conceptual development of McCombs and Guo (2014); Schroeder (2015, 2018); Li et al. (2024); Schlosberg (2016); Jungherr and Schroeder (2023).

As delinated in Table 1, these indicators demonstrate that digital political contestation is no longer merely an ideological struggle, but a highly rationalized, bureaucratic procedure. Elites do not simply persuade; they weaponize the cognitive void left by the secondary digital divide to capture the attention space, transforming democratic voting behavior into a highly predictable outcome of digital engineering.

### The Indonesian digital divide: causal mechanisms of elite domination

To understand how political elites weaponize this digital bureaucracy in Indonesia, the country’s macro-structural data must be analyzed through the causal lens of stratified digital capital. While global digital divides historically focused on raw infrastructural access—such as the massive offline populations in India (730 million) and China (375 million)—Indonesia presents a distinct structural paradox (Schroeder, 2015; We Are Social and Hootsuite, 2023). According to Kemp (2024), Indonesia ranks 8th globally in terms of unconnected populations, with 63.5 million citizens lacking internet access. Yet, simultaneously, the nation boasts a hyper-connected urban–rural core comprising 185.4 million active internet users and 139.0 million active social media consumers, representing 49.9% of the total population (see Table 2).

**Table 2:** Active users of social media platforms in Indonesia in 2024.

Social media platform	Number of active users (millions)
Youtube	139.0
Tiktok	126.8
Facebook	117.6
Instagram	100.9
LinkedIn	26
X (formerly Twitter)	24.69
Snapchat	2.05

Source: (Kemp, 2024).

The causal link between this hyper-connectivity and elite dominance becomes visible when we evaluate the secondary digital divide: the severe stratification of digital literacy and cognitive competence across these user bases. Data from the Reuters Institute underscores that social media platforms are highly stratified by educational background; users with higher education levels are disproportionately clustered on text-heavy, elite-dominated spaces like X (Twitter), while mass populations with secondary or lower educational backgrounds heavily saturate visual-centric, algorithmic platforms like TikTok, Facebook, and Instagram (Santika, 2023).

This stratification interacts directly with Indonesia’s critically low national digital literacy index, which stands at a meager 3.54 out of 5.00, rendering the country the lowest in ASEAN with an average competency rate of only 62% compared to the 70% regional baseline (Directorate General of Informatics Applications, 2022; Anam, 2023). Operating at what Ragnedda and Kreitem (2018) define as the first and second levels of the digital divide (access without structural competence), the Indonesian populace possesses the technical capacity to navigate networks but lacks the semantic capital to critically evaluate digital content (Paterson, 2019; Sabrina, 2019; Ahmad, 2022).

From a Weberian perspective, this deficit in digital competence creates a profound cognitive vacuum. Because users lack the tools for substantive rational validation, they default to the formal, procedural validation provided by the platform’s metrics (e.g., view counts, viral status, algorithmic trend placement). Political elites systematically exploit this structural vulnerability by introducing “digital pests”—such as institutionalized misinformation, state-aligned hoaxes, and coordinated disinformation campaigns (Farkas and Schou, 2018; Prayoga, 2022; Konieczny, 2023). The crisis is further intensified by chronic structural vulnerabilities in state digital infrastructure, where massive personal data breaches place Indonesia 8th globally in data exposure (Ahdiat, 2024; Prasetyo, 2024). This structural insecurity effectively strips the public of data sovereignty, transforming private demographics into raw material for elite algorithmic targeting. In this post-truth era, as conceptualized by Reed (2018), technological sophistication is deployed not to inform, but to distort dominant belief systems. By destabilizing objective frameworks of validity, digital media elites ensure that “right can appear wrong, and wrong can appear right,” cementing a form of digital domination where public rationality is structurally subverted by engineered algorithmic architectures.

### Political contestation and the attention space: empirical evidence from the 2024 “Gemoy” campaign

The empirical manifestation of this digital bureaucracy and engineered domination was clearly captured during our systematic digital observation of the 2024 Indonesian Presidential Election. As noted by Shcheglova (2022), contemporary digitalization alters deeply ingrained behavioral and cultural preferences, compressing space and time to bind populations within localized, high-retention attention environments. During the six-month observation window (October 2023–March 2024), political elites actively bypassed traditional institutional media gatekeepers to conquer this compressed public “attention space,” transforming complex democratic discourse into a highly calculated aesthetic commodity (Schroeder, 2015).

**Figure 2:**
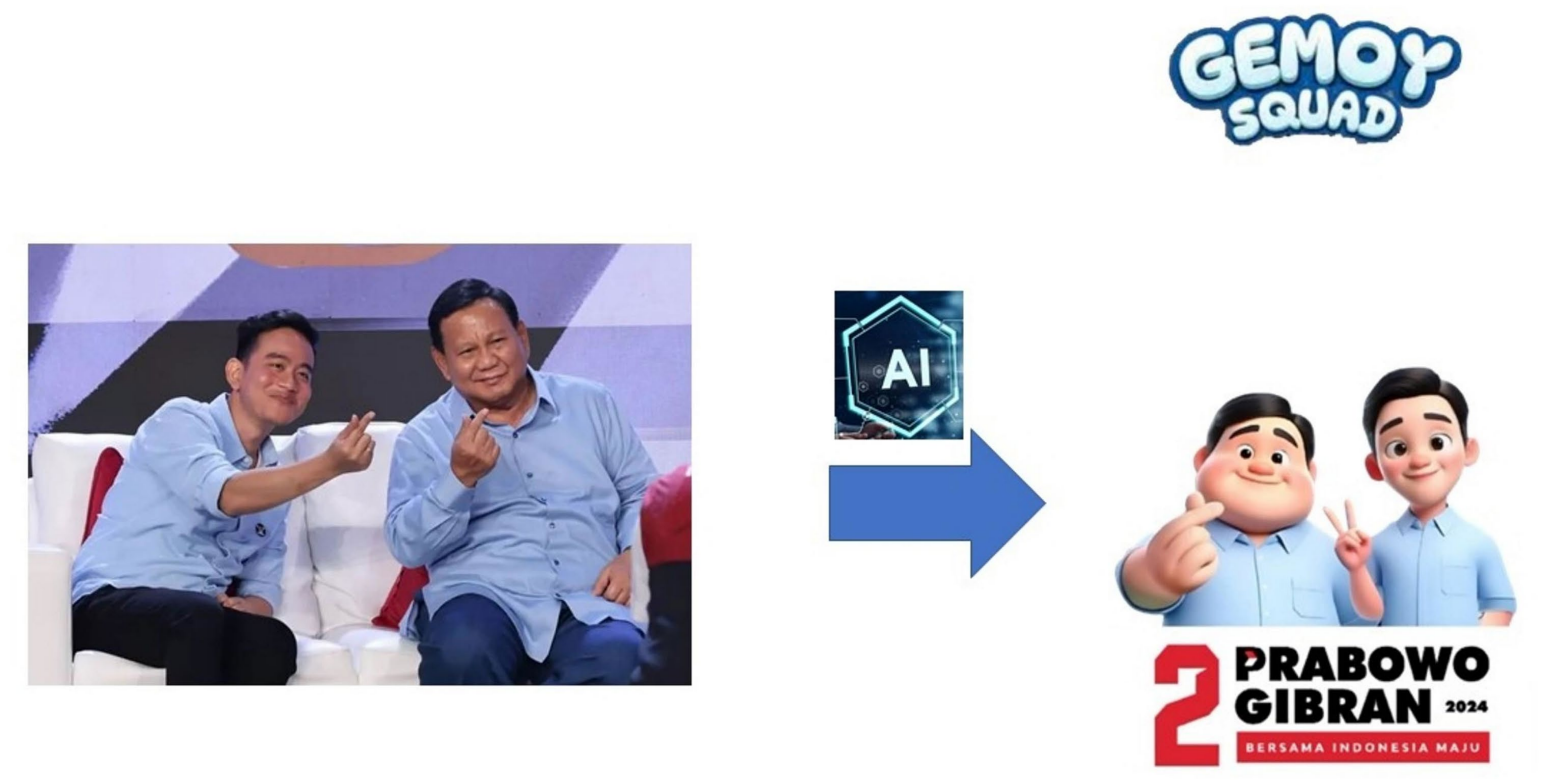
AI-generated transformation of Prabowo Subianto and Gibran Rakabuming Raka into the “Gemoy” avatar. Left photo reproduced from Radar Kudus / Jawa Pos (https://radarkudus.jawapos.com/nasional/693341269/gemoy-bukan-sekadar-gimik-prabowo-bawa-program-buat-generasi-muda-tertarik). Right AI avatar reproduced from Twibbonize (https://www.twibbonize.com/gemoysquad).

The structural execution of this dynamic is empirically demonstrated by the Prabowo_Gibran campaign’s strategic deployment of the AI-generated “Gemoy” avatar. Our systematic monitoring and axial coding of observational logs from TikTok, Instagram, and X reveal that the campaign did not merely use digital media as a tool for message dissemination; rather, it actively engineered an entirely synthetic political ecosystem. The campaing systematically withdrew from traditional rational-legal political debates—such as policy position papers or ideological disputation and instead saturated visual-centric platforms with highly optimized, synthetic imagery. As shown in Figure 2, the transformation of raw political figures into non-threatening, cute, AI-designed cartoon avatars effectively re-engineered the candidates’ public identities (Anugerah, 2024; Ginasari et al., 2024).

Analyzed through Weber’s theory of political authority, our empirical data indicates that the “Gemoy” phenomenon represents a highly modernized, simulated variant of plebiscitary charismatic domination *(plebiszitüre Führerdemokratie)*. In Weber’s classic plebiscite model, the masses effectively surrender active democratic inputs, acting merely as a passive apparatus that validates a leader through highly staged, emotional mechanisms. In the digital era, our observational evidence shows that this charisma is no longer organic; it is algorithmically engineered and synthetically manufactured via machine learning software to deliberately target stratified demographics (Masaaki and Akihiro, 2022; Armiwulan et al., 2024).

To demonstrate the systematic nature of this digital domination, our thematic analysis organized the observational data into three highly structured empirical mechanisms: First, Algorithmic Satiation, our tracking of viral political hashtags and feed trends during the campaign peak demonstrated that the AI-generated “Gemoy” content was structurally amplified by coordinated platform algorithms. This automated saturation created a dense digital echo chamber that systematically dominated the attention span of Gen Z and millennial voters, who constituted over 50% of the total electorate (Masaaki and Akihiro, 2022; Anugerah, 2024). The sheer volume of synthetic content functionally blocked alternative political narratives from entering the users’ primary feed spaces, confirming Schroeder (2015) premise regarding the asymmetric control of digital content density. Second, Cognitive Desensitization, through the systematic coding of public engagement within the comment sections of elite account, our data revealed a profound shift from political evaluation to asthetic consumption. Substantive political questions regarding human right, economic ineqaulity, state transparency, and digital exploitation were structurally drowned out by a homogeneus wave of aesthetic praise for the avatar’s “cuteness.” The visual format of the avatar functioned as an emotional armor, rendering traditional rational-legal critiques obsolete and demonstrating how formal platform aesthetic can successfully subvert substantive societal rationality. Third, Procedural Validation, our observation logs captured the exact transition where a hyper-connected yet low-literate audience was transformed into a functional extension of the elite’s political machine. Rather than acting as critical democratic agents, users voluntarily reproduced, liked, commented, and redistributed the template content. By participating in these standardized platform procedures, the audience voluntarily entered the digital “iron cage,” converting synthetic algorithmic visibility into real, mobilized ballot box compliance.

Ultimately, the empirical data from the 2024 election proves that digital media in Indonesia fusntions precisely as a rationalized, bureaucratic tool of domination. By controlling the digital content flow and capitalizing on structural literacy deficits, political elites did not engage public rationality—they methodically bypassed it, sucessfully manufacturing collective public trust and political compliance through automated procedural mechanisms.

## Discussion

The empirical findings of this study demonstrate that the digital divide in Indonesia has evolved far beyond a technical constraint of infrastructural access into a highly strategic mechanism of elite political domination. By synthesizing our observations of the 2024 Presidential Election with Schroeder (2015) comparative media systems framework, this study Moves beyond the mere application of classical sociology. Instead, it offers distinct theoretical advancements to contemporary digital sociology by refining how authority, attention spaces, and rationalization operate within hyper-connected yet low-literacy context.

### Beyond organic Charisma: conceptualizing “algorithmic Charisma”

The primary theoretical contribution of this article lies in its refinement of Max Weber’s classic typology authority, specifically concerning charismatic domination *(Charismatische Cerrschaft).* Traditional political communication literature often attributes populist electoral victories to organic, innate charisma or direct, unmediated grassroots mobilization. However, our empirical analysis of the “Gemoy” campaign during the 2024 Indonesia Presidential Election challenges this conventional assumption. Our finding demonstrate that in contemporary digital political landscapes, charisma is no longer purely personal or inherently disruptive. Instead, as synthesized from our digital observation, charisma can be synthetically manufactured, algorithmically curated, and procedurally sustained through artificial intelligence and automated platform engineering. We define this phenomenon as “Algorithmic Charisma”—a highly calculated, aesthetic political persona designed to capture the public attention space. When political figures ate repackaged as AI-generated avatars, the visual cuteness systematically function as an emotional shield that immunizes candidate from rational-legal accountability and structural critiques regarding human right or economic inequality. This proves that in the digital era, bureaucracy (in the form of platform algorithms) no longer neutralizes charisma; rather, it has become the primary mechanism through which charisma is engineered to manufacture public compliance.

### The participatory iron cage: redefining digital bureaucracy

Furthermore, this study extend the scope of the “attention space” theory and the Weberian concept of the “iron cage” (*Stahlhartes Gehäuse*) of rationalization. While Schroeder (2018) points out that digital media inherently shifts content choice toward the user, granting them greater autonomy, our analysis reveals that this perceived autonomy is structurally superficial. In enviroments characterized by a severe gap in digital literacy, such as Indonesia’s national indeks of 3.54, the public’s interaction with the attention space is governed not by value-rational deliberation, but by automated algorithmic satiation. This dynamic establishes what we term a “Participatory Iron Cage.” Unlike traditional top-down authoritarian censorship, digital media elites in Indonesia construct automated environments—or digital bureucracies—that process and standardize content to manipulate public perception. Driven by the formal rationality of the platform (e.g., metric maximization, viral trends, and algorithmic feed placement), the hyper-connected audience does not resist this control. Instead, they voluntarily and enthusiastically participate in their own subordination by liking, commenting on, and redistributing elite-driven content, effetively converting algorithmic engagement into formal political validation at the ballot box.

### Repositioning the digital divide in political sociology

Finally, this study challenges the traditional boundaries of the digital divide literature by grounding its critique in the framework established by Ragnedda and Kreitem (2018). By operating explicitly at what Ragnedda and Kreitem (2018) define as the secondary digital divide—characterized by access to networks without structural, cognitive, or semantic competence—the indonesian case illustrates how cognitive vulnerabilities are actively converted into political capital. In a post-truth era increasingly plagued by chronic data breaches and systemic misinformation, the incapacity to critically evaluate digital truth does not merely result in socio-economic marginalization; it facilitates a subtle, highly rationalized form of digital authoritarianism where “right can appear wrong, and wrong can appear right.” This critical insight repositions the digital divide from a simple, descriptive metric of technological inequality into a primary, structural weapon of elite hegemony within transitional democracies.

## Conclusion

This study concludes that the rapid expansion of internet connectivity and social media usage in Indonesia has failed to democratize the political public sphere, primarily due to persistent structural gaps in the secondary digital divide. While technical access is widespread, Indonesia’s low national digital literacy creates a critical cognitive vulnerability. Rather than fostering open democratic deliberation, this environment enables political elites to bypass traditional journalistic gatekeepers and systematically conquer the public attention space. Through the strategic deployment of AI-generated content during the 2024 Presidential Election—exemplified by the “Gemoy” campaign—digital media has been effectively transformed into an automated digital bureaucracy that operates through three verified empirical mechanisms: algorithmic satiation, cognitive desensitization, and procedural validation. Theoretically, this research advances contemporary Weberian sociology by introducing two novel conceptual frameworks. First, it conceptualizes “Algorithmic Charisma,” proving that in the digital era, emotional political authority is no longer purely organic or personally disruptive, but can be synthetically engineered via artificial intelligence to insulate elites from rational-legal scrutiny. Second, it formulates the “Participatory Iron Cage,” illustrating how hyper-connected audiences, driven by the formal rationality of platform metrics, voluntarily reproduce elite-driven content, converting algorithmic engagement into formal electoral compliance. Ultimately, this study repositions the digital divide within global political sociology. In transitional democracies across the Global South, structural deficits in digital competence do not merely reflect socio-economic inequality; they serve as a primary, rationalized weapon of elite domination that transforms a hyper-connected electorate into passive participants within engineered procedural mechanisms.

### Limitations and future research

Despite its structural insights, this study possesses certain limitations that should be addressed in subsequent scholarship. First, the digital observation was bounded by a specific six-month window surrounding the 2024 Indonesian Presidential Election and localized primarily on X (formerly Twitter) and Instagram. Consequently, the findings may not fully capture the long-term, daily operational strategies of digital elites outside of high-stakes electoral cycles, nor do they account for the distinct algorithmic architectures of other rapidly growing platforms like TikTok or WhatsApp closed-loop networks. Second, as a qualitative study rooted in thematic analysis and Weberian deductive interpretation, this research lacks large-scale computational data tracking, such as social network analysis (SNA) or automated sentiment scraping, which could provide a quantitative map of elite bot networks and algorithmic propagation speeds.

Future research should therefore employ mixed-method approaches that integrate computational sociology with in-depth digital ethnography to track the precise mechanics of algorithmic manipulation across diverse political events. Furthermore, comparative studies across other Southeast Asian transitional democracies are highly recommended to evaluate whether the weaponization of low digital literacy is a unique localized phenomenon or a broader systemic trend defining the future of digital politics in the Global South.

## Data Availability

The raw data supporting the conclusions of this article will be made available by the authors, without undue reservation.
